# A pilot genome-wide association study of ischemic heart disease with co-occurring arterial hypertension in a Kazakh cohort

**DOI:** 10.3389/fgene.2026.1927147

**Published:** 2026-10-01

**Authors:** Liliya Skvortsova, Kanagat Yergali, Assel Zhaxylykova, Mamura Begmanova, Almagul Mansharipova

**Affiliations:** 1 Laboratory of Molecular Genetics, Institute of Genetics and Physiology, Almaty, Kazakhstan; 2 Department of General Medical Practice, Kazakh-Russian Medical University, Almaty, Kazakhstan

**Keywords:** arterial hypertension, Central Asia, genome-wide association study, ischemic heart disease, Kazakh population, ZFHX3

## Abstract

**Background:**

Genome-wide association studies (GWAS) of ischemic heart disease (IHD) remain underrepresented in Central Asian populations. We conducted a pilot GWAS of ischemic heart disease with co-occurring arterial hypertension (IHD-HTN) in an ethnic Kazakh cohort to identify candidate loci for future replication.

**Methods:**

This study included 470 Kazakh individuals, comprising of 240 cases and 230 controls genotyped using the Illumina Infinium Global Screening Array-24 v 3.0. Association testing was performed using logistic regression under an additive genetic model adjusted for age, sex, and the first 10 principal components. Post-imputation GWAS was performed using high-quality imputed variants. Secondary analyses included MAGMA and KGWAS prioritization.

**Results:**

In the array-based GWAS, no variant reached the Bonferroni-corrected significance threshold (P < 1.53 × 10^−7^). The strongest SNP association was observed for rs1511924 at chromosome 16 mapped to the ZFHX3 gene (OR = 0.49, 95% CI = 0.37–0.66; P = 1.00 × 10^−6^). Post-imputation GWAS supported the same locus with rs12598508 (P = 4.93 × 10^−7^) and rs1511924 (P = 5.15 × 10^−7^) among the strongest signals, although neither reached the genome-wide significance. Secondary MAGMA and KGWAS analyses did not provide stable evidence for additional loci.

**Conclusion:**

To our knowledge, this is the first pilot GWAS focused on IHD with co-occurring arterial hypertension in a Kazakh cohort. Although no Bonferroni-significant SNP associations were identified, the ZFHX3 region emerged as the strongest suggestive candidate locus and requires independent replication. Larger Central Asian cohorts and population-specific reference resources are required to validate these findings.

## 1 Introduction

Ischemic heart disease (IHD) remains a leading cause of death and disability worldwide (Shi et al., 2025). Although global IHD mortality has decreased since 2000, recent analyses by World Health Organization (WHO) show uneven trends with persistent or increasing rates in parts of Asia, Africa, and Central/South America. Kazakhstan has relatively high but gradually decreasing age-standardized mortality; nevertheless, cardiovascular diseases and IHD remain the country’s leading causes of death (Mukasheva et al., 2022; Jaiswal et al., 2025). National data indicate that IHD mortality decreased in 2011–2019 but increased again in 2020–2021, coinciding with the COVID-19 pandemic and disruption of cardiovascular morbidity and mortality patterns (Zhakhina et al., 2023; Kosherbayeva et al., 2024). Because IHD develops through long-term exposure to modifiable risk factors, such as obesity, smoking, and physical activity, on a background of inherited susceptibility, genetic predisposition remains an important component of the risk. Family history of premature IHD is an independent risk factor, and twin studies support substantial heritability (Ozaki and Tanaka, 2016; Miyazawa and Ito, 2021).

Large genome-wide association studies (GWAS) and meta-analyses including CARDIoGRAMplusC4D consortia and UK Biobank studies have identified hundreds of loci associated with IHD and myocardial infarction, confirming the polygenic nature of the disease and enabling polygenic risk prediction and therapeutic target discovery (Nelson et al., 2015; Tcheandjieu et al., 2022; Kessler and Schunkert, 2021; Miyazawa and Ito, 2021; Matsunaga et al., 2020). However, most IHD GWASs have been conducted in European ancestry populations, with limited Asian representation and almost no genome-wide IHD data from Central Asia. Public reference panels including 1,000 Genomes and gnomAD contain no or very few Central Asian individuals. It should be noted that few sequencing studies of Kazakhs indicate mixed European and East Asian ancestry and substantial heterogeneity (Seidualy et al., 2020; Karczewski et al., 2020; Fatumo et al., 2022; Akilzhanova et al., 2014; Kairov et al., 2021; Kairov et al., 2022). Substantial genetic heterogeneity underscores the need for population-specific genomic data from this region (Narasimhan et al., 2019).

Disease-focused genetic studies of IHD in Kazakhstan remain limited. Most available studies have used candidate gene approaches and examined selected polymorphisms in the renin–angiotensin system, coagulation and inflammatory genes, 9p21.3 (CDKN2B-AS1/ANRIL), loci related to coronary restenosis, coronary artery disease in type 2 diabetes and cardiac autonomic neuropathy, hypertension, stroke, and oxidative stress-related loci (Baĭtasova and Rysmendiev, 2002; Aitkhozhina and Lyudvikova, 2003; Myngbay and Alibekov, 2023; Aitkaliyev et al., 2024; Taizhanova et al., 2020; Taizhanova et al., 2023; Shakhanova et al., 2020; Bekenova et al., 2024; Aitkaliyev et al., 2024; Muratbekova et al., 2025; Svyatova et al., 2023). In our previous case–control study, we assessed GCLM -588C/T and GCLC -129T/C promoter polymorphisms in relation to IHD development in the population of Kazakhstan (Skvortsova et al., 2017). These studies provided initial evidence on cardiovascular genetic risk in this underrepresented population. However, they covered only a limited number of loci and cannot characterize the genome-wide architecture of IHD in Kazakhs.

More comprehensive genomic approaches are therefore needed. Whole-genome and whole-exome sequencing can provide detailed variant level data, but their cost limits their use in large Kazakhstani cohorts. In contrast, high-density SNP array-based GWAS is more feasible population-scale approach for detecting common IHD risk variants. Therefore, we conducted a pilot GWAS of IHD with co-occurring arterial hypertension in a population of ethnic Kazakhs (1) to identify genetic variants associated with the combined phenotype of IHD and hypertension, (2) to examine whether these loci previously implicated in IHD or hypertension in other ancestries, and (3) to explore potential population-specific signals that warrant replication in larger cohorts.

## 2 Materials and methods

### 2.1 Ethics approval, biosamples, and consent to participate

The study included a combination of archived and prospectively collected biosamples. The use of archived samples for genetic research was approved by the Local Ethics Committee of Kazakh-Russian Medical University, Almaty, Kazakhstan (Approval No. 36, 1 May 2016). Archived samples and/or associated clinical records were accessed for the research on 3 February 2025. Prospectively collected samples were obtained from 5 January 2024 to 30 August 2025 under approval from the Local Ethics Committee of the Institute of Genetics and Physiology, Almaty, Kazakhstan (Protocol No. 3, 30 October 2023; excerpt letter No. 13–368, 17 November 2023).

The study complied with the Declaration of Helsinki and applicable national regulations. Written informed consent was obtained from all prospectively enrolled participants. The use of archived samples was authorized under the applicable ethics approval and consent procedures in place at the time of collection.

### 2.2 Study subjects

Peripheral blood samples and clinical data were collected from participants recruited through City Clinical Hospital No. 1 and Kazakh-Russian Medical University in Almaty, Kazakhstan. Peripheral blood was obtained as a research biospecimen in accordance with the corresponding ethics-approved study procedures. Both case and control participants were restricted to the age range of 50–70 years. Patients with a clinically defined combined phenotype of IHD with co-occurring arterial hypertension (IHD-HTN), representing a clinically relevant cardiovascular multimorbidity pattern, were recruited for the study. The case group included patients with clinically documented IHD-HTN including stable angina pectoris (Canadian Cardiovascular Society classes I–IV and Association of Cardiologists of the Republic of Kazakhstan (Campeau, 1976)), chronic heart failure (New York Heart Association classes II–III) (Nomenclature and Criteria for Diagnosis of Ischemic Heart Disease, 1979), and post-infarction cardiosclerosis, hypertension, and current use of antihypertensive medication. IHD diagnosis was based on documented clinical assessment performed during routine medical care in accordance with the WHO criteria. According to the available medical records, diagnostic evaluation included assessment of chest pain symptoms, electrocardiography (ECG), exercise testing, and/or coronary angiography and cardiac biomarker measurements (troponin, myoglobin, and creatine kinase). Exclusion criteria were inflammatory or metabolic status including diabetes, inflammatory diseases, local inflammatory processes, allergic reactions, signs of renal insufficiency, circulatory insufficiency of stage III, Alzheimer’s disease, presence of hyperthermia, and insolation during the previous 2–3 weeks.

The control group included apparently healthy participants without clinical manifestations of IHD and arterial hypertension, oncology, autoimmune diseases, known hereditary diseases, and acute/chronic inflammatory diseases. Demographic, lifestyle, self-reported ethnicity, and medical history information was obtained using standardized questionnaires routinely used at the Institute of Genetics and Physiology and stored in the study metadata database for both archived and prospectively collected biosamples.

### 2.3 Genomic DNA isolation

All archived and prospectively collected specimens were processed within the present project using the same laboratory procedures. DNA was extracted from EDTA-treated peripheral blood samples using the « ReliaPrep™ Blood gDNA Miniprep System» (Promega, USA). Qualitative and quantitative characteristics of the extracted DNA samples were estimated using spectrophotometry.

### 2.4 Genotyping and quality control

Genomic DNA samples extracted from archived and prospectively collected peripheral blood specimens were genotyped *de novo* within the present study using the Illumina Global Screening Array-24 v3.0 BeadChip. Raw IDAT intensity files from all genotyping runs were imported into a single GenomeStudio project, and genotype clustering and calling were performed jointly across the complete study cohort using the GenomeStudio Genotyping Module v2.0, rather than separately, according to sample provenance or phenotype status. Genotypes were subsequently exported in Illumina A/B allele format with marker annotations. Sample quality control (QC) excluded individuals with a call rate <0.95, sex discordance, or unresolved duplicate status. Variant QC retained autosomal SNPs with a call rate ≥0.98, a minor allele frequency ≥0.03, and Hardy–Weinberg equilibrium in controls at P ≥ 1 × 10^−6^. Sex chromosome and XY-region variants were excluded.

### 2.5 Statistical analysis

#### 2.5.1 Clinical statistics

Baseline clinical characteristics were summarized as mean ± standard deviation (SD) for continuous variables and as counts (percentage) for categorical variables. Comparisons between case and control groups were performed using Student’s t-test for independent samples for continuous variables, including age and systolic/diastolic blood pressure. The presence of arterial hypertension was considered a categorical variable and compared between groups using the chi-square test or Fisher’s exact test as appropriate. All statistical tests were two-sided.

#### 2.5.2 Linkage disequilibrium pruning and SNP selection for principal component analysis

Prior to the principal component analysis (PCA), autosomal SNPs located within known long-range linkage disequilibrium (LD) regions for the GRCh37/hg19 genome build, including the major histocompatibility complex (MHC) on chromosome 6, were excluded to reduce the influence of extended LD on population structure inference (Price et al., 2008). Illumina A/B genotype calls were converted to numeric B-allele dosage coded as 0, 1, or 2, while missing or non-informative genotype calls were encoded as missing. Genome-wide LD pruning was then performed in Python using a PLINK-like sliding-window approach analogous to - - indep-pairwise with SNPs ordered by chromosomal position and evaluated in 50-SNP windows advanced by five SNPs at each step (Purcell et al., 2007; Chang et al., 2015). Pairwise LD was quantified using the squared Pearson correlation coefficient (*r* ^2^), and one SNP from each pair with *r* ^2^ > 0.2 was removed. The resulting approximately LD-independent set of autosomal markers was used for PCA-based assessment of population structure (Price et al., 2006; Patterson et al., 2006; Price et al., 2006).

#### 2.5.3 Principal component analysis

Population structure was assessed independently of self-reported ethnicity by PCA using the LD-pruned autosomal SNP set. Genotypes were represented as Illumina B-allele dosages coded as 0, 1, or 2. After transposition to a (samples × SNPs) matrix, missing genotypes were imputed with the SNP-specific mean dosage, and genotypes were standardized per SNP. PCA was performed by singular value decomposition of the standardized matrix (Patterson et al., 2006; Harris et al., 2020). The first 10 principal components were retained for visualization and as covariates in the GWAS models. One outlier-removal step was performed based on the initial PC1−PC2 distribution, and PCA was recomputed in the remaining individuals before final association testing (Price et al., 2006).

#### 2.5.4 Linkage disequilibrium decay analysis

Genome-wide LD decay was compared between the Kazakh study cohort and European (EUR), East Asian (EAS), and South Asian (SAS) reference populations from the 1000 Genomes Project Phase 3. Analyses were performed on harmonized autosomal SNPs shared across all populations. For the primary comparison, variants with minor allele frequency (MAF) ≥0.05 within each population were retained, followed by restriction to the common intersecting SNP set across KAZ, EUR, EAS, and SAS. Pairwise LD was calculated (*r* ^2^) using PLINK v1.9 for SNP pairs located within 1 Mb without prior LD pruning. Physical distances were grouped into nine intervals (0–1, 1–5, 5–10, 10–25, 25–50, 50–100, 100–250, 250–500, and 500–1,000 kb), and mean and median pairwise (*r* ^2^) were calculated for each interval. Sensitivity analyses were performed using MAF thresholds of ≥0.01 and ≥0.03 following the same procedure.

#### 2.5.5 Genotype imputation

Genotype imputation was performed using the TOPMed Imputation Server with hg19/GRCh37 input files, Eagle phasing, and the TOPMed r3 reference panel. Post-imputation quality control retained autosomal biallelic variants with imputation quality *R* ^2^ ≥ 0.8 and MAF ≥ 0.01.

#### 2.5.6 Genome-wide association analysis

GWAS was performed using Python 3.12.12. Genotype dosage was coded as 0, 1, or 2, corresponding to the number of Illumina B alleles. Disease status was coded as 1 for cases and 0 for controls. Logistic regression was performed under an additive genetic model adjusted for age, sex, and the first 10 genetic principal components. Odds ratios (ORs) were calculated as exp(β) and represent the per B-allele effect; 95% confidence intervals were derived from the standard errors of the genotype coefficients. Variants with non-convergent or unstable regression estimates were reported with missing association statistics. Minor allele frequency was calculated as min (fA,fB) from Illumina A/B allele frequencies and therefore did not necessarily correspond to the effect allele frequency. The MAF threshold was applied during the QC procedure using allele-frequency values rounded to two decimal places. Illumina A/B alleles were converted to forward-strand nucleotide alleles using the GSA-24v3-0_A1 Strand Report.

The post-imputation GWAS was performed in PLINK2 using imputed allele dosages rather than discrete genotype calls coded as 0, 1, or 2. Dosage values were imported from the post-imputation dosage VCF files and represented the expected allele count on a continuous scale from 0 to 2. Association testing was performed under an additive logistic regression model adjusted for the same covariates (age, sex, and PC1–PC10). The effect allele was defined as the tested allele reported in the PLINK2 A1 field, and β coefficients and ORs therefore represent the effect per additional copy of this allele. Only autosomal biallelic variants passing post-imputation quality control (*R* ^2^ ≥ 0.8 and MAF ≥ 0.01) were included in the analysis.

For both analyses, statistical significance thresholds were determined according to the number of variants tested, and genomic inflation (λGC) was calculated from the ratio of the observed to expected median chi-square statistic.

#### 2.5.7 Variant annotation

For the array-based GWAS results, annotation of variants to genes was performed using GENCODE v19 annotation based on the GRCh37/hg19 genome assembly (Harrow et al., 2012). SNP positional mapping and nearest gene assignment were performed using gene coordinates from this annotation. Gene identifiers were subsequently converted to gene symbols and gene names using the MyGene.info API (MyGeneInfo Python interface; *Homo sapiens*; fields: Entrez gene identifier, gene symbol, and gene name) (Wu et al., 2013). Prioritized variants were additionally manually checked using the UCSC Genome Browser (GRCh37/hg19) based on rs identifiers (Casper et al., 2026).

For post-imputation regional visualization, gene models were obtained from GENCODE v50 annotation based on the GRCh38/hg38 assembly (Frankish et al., 2019).

#### 2.5.8 Knowledge graph-guided and gene level analyses of GWAS summary statistics

Exploratory secondary analyses were performed using conventional MAGMA and KGWAS (de Leeuw et al., 2015; Huang et al., 2024). Prior to these secondary analyses, GWAS summary statistics underwent additional harmonization to remove non-unique or otherwise incompatible variant records and to retain variants suitable for downstream SNP-to-gene mapping and tool-specific matching. MAGMA v1.10 was applied to array-based and post-imputation GWAS SNP p-values in SNP-wise mean mode using GRCh37/hg19 coordinates with SNPs mapped to genes by genomic position. Given the limited availability of public Kazakh LD reference panels, MAGMA analyses used our in-sample custom Kazakh LD reference panel and 1000 Genomes Phase 3 East Asian and European panels as comparative ancestry-sensitivity references. Bonferroni-corrected gene level thresholds were calculated separately for each LD reference analysis. KGWAS was run using the official implementation and precompiled functional genomics knowledge graph with variants matched by rsID. KGWAS analysis was performed for both array-based and post-imputation GWAS summary statistics, and repeated training runs were used to assess the stability of SNP prioritization.

## 3 Results

### 3.1 Cohort characteristics

Initially, 509 individuals were recruited and genotyped. After GWAS-specific QC, the final dataset included 470 self-identified Kazakh individuals: 240 IHD-HTN cases and 230 apparently healthy controls. The sample QC workflow is provided in Supplementary Table S1. Baseline characteristics are shown in Table 1.

**TABLE 1:** Baseline characteristics of the final GWAS cohort after sample quality control.

Variable	Cases (*n* = 240)	Controls (*n* = 230)	*p*-value
Age, years (mean ± SD)	56.7 ± 7.3	54.6 ± 6.2	0.0008
Sex, male n (%)	110 (45.8)	95 (41.3)	0.326
SBP, mmHg (mean ± SD)	149.5 ± 14	119.1 ± 3.5	<0.001
DBP, mmHg (mean ± SD)	94.1 ± 14.5	60 ± 11.2	<0.001
Hypertension diagnosis, n (%)	240 (100.0)	0 (0.0)	−
Ethnicity	Kazakh	Kazakh	−
Smoking status	​	​	0.016
Smokers, *n* (%)	57 (23.8)	33 (14.3)	​
Non-smokers, n (%)	9 (3.8)	16 (6.9)	​
Unknown, *n* (%)	174 (72.5)	181 (78.7)	​

Cases were statistically older than controls (56.7 ± 7.3 years in cases and 54.6 ± 6.2 years in controls, p = 0.0008, Cohen’s d = 0.31), whereas sex distribution did not differ significantly between groups (male 45.8% vs. 41.3%, p = 0.326). As expected from the phenotype definition, blood pressure values were higher in the cases than in the controls, with mean systolic blood pressure (SBP) of 149.5 ± 14 mmHg vs. 119.1 ± 3.5 mmHg and mean diastolic blood pressure (DBP) of 94.1 ± 14.5 mmHg vs. 60 ± 11.2 mmHg (both p < 1 × 10^−6^). Smoking status differed between the groups (χ^2^ test, p = 0.016), but this variable was not included in the primary GWAS model because smoking information was missing for the most participants.

### 3.2 Principal component and population structure analysis

Principal component analysis was used to evaluate genetic variation within the study cohort and to place the Kazakh samples in a broader population context. In the within-cohort analysis, PCA was performed using LD-pruned autosomal SNPs from the 470 study participants. The PC1–PC2 plot showed substantial overlap between IHD-HTN cases and controls, with no evident clustering related to disease status (Supplementary Figure S1A).

To further characterize the genetic background of the cohort, we performed a joint PCA including the Kazakh samples and 1000 Genomes Phase 3 reference populations representing European (EUR), East Asian (EAS), and South Asian (SAS) groups. Based on shared harmonized autosomal SNPs, Kazakh individuals showed a broad distribution within the Eurasian genetic space, with positions overlapping or located close to European and East Asian reference populations and intermediate positions between these groups (Supplementary Figure S1B).

Overall, the PCA results indicated variation in genetic background among the studied Kazakh individuals, while the distribution of cases and controls remained largely overlapping within the cohort.

Genome-wide LD decay showed a broadly similar distance-dependent pattern across the KAZ, EUR, EAS, and SAS groups, with strong LD at short genomic distances followed by progressive decay toward low background levels (Supplementary Figure S2). In the primary analysis restricted to variants with MAF ≥0.05, EAS showed slightly higher mean (*r* ^2^) at short distances, whereas the Kazakh cohort generally showed median (*r* ^2^) values at or near the upper range of the reference populations. Differences between populations became smaller with increasing physical distance, although the Kazakh cohort retained modestly higher median (*r* ^2^) at distances above approximately 100 kb. Sensitivity analyses using MAF thresholds of ≥0.01 and ≥0.03 showed the same overall qualitative pattern.

### 3.3 Genome-wide association analysis

A total of 325,976 array-based and post-QC autosomal SNPs were included in the association analysis. The detailed sample provenance by case/control status and SNP-level QC workflow is summarized in Supplementary Tables S1, S2. The Bonferroni-corrected significance threshold was P < 1.53 × 10^−7^. No variant met this threshold (Figure 1A). The strongest SNP association signal was observed for rs1511924 at chromosome 16 mapped to the ZFHX3 locus. The effect allele C showed a protective direction of the effect (OR = 0.49, 95% CI = 0.37–0.66; p = 1.00 × 10^−6^). This signal was further supported by the post-imputation GWAS of 6,668,741 variants in which the strongest associations mapped to the ZFHX3 region including rs12598508 (p = 4.93 × 10^−7^) and rs1511924 (p = 5.15 × 10^−7^) (Figure 1B). Although neither variant reached the Bonferroni-corrected or conventional GWAS significance thresholds, regional LD analysis demonstrated near-complete correlation between rs12598508 and rs1511924 (*r* ^2^ = 0.997), providing additional non-independent regional support for the ZFHX3 candidate locus (Supplementary Figure S3). Additional top-ranked variants for both analyses are summarized in Table 2. The QQ plots for both the array-based and post-imputation analyses showed close agreement between observed and expected p-value distributions, indicating no substantial inflation of association statistics (λ_GC_ = 1.046 and 1.048, respectively).

**FIGURE 1:**
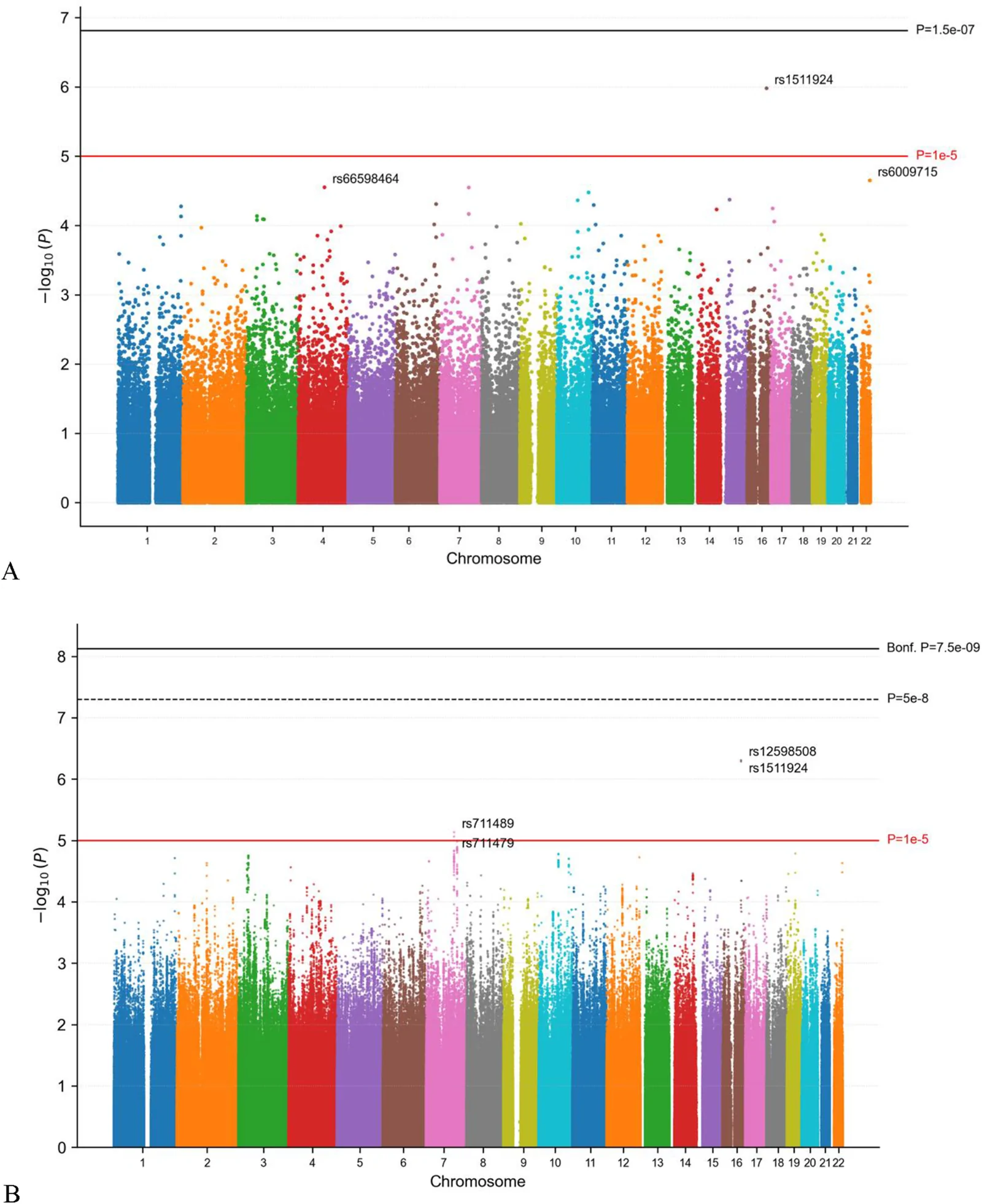
Manhattan plots of the GWAS results for IHD-HTN before and after genotype imputation. **(A)** GWAS results based on directly genotyped SNPs. **(B)** post-imputation GWAS results based on variants retained after imputation quality control. Each point represents an SNP plotted by chromosomal position with the y-axis showing −log_10_(P). The horizontal black line indicates the Bonferroni-corrected significance threshold, and the red line indicates a suggestive threshold (p = 1 × 10^−5^). In panel **(B)**, the dashed black line additionally indicates the conventional genome-wide significance threshold (p = 5 × 10^−8^). The most significant variants are annotated using the rsID.

**TABLE 2:** Top associated SNP variants from the GWAS.

No	RsID	Chr	Position (GRCh37)	Gene annotation	Functional context	Effect allele	Other allele	OR for the effect allele	*p*-value	EAF
Top variants from the array-based GWAS										
1	rs1511924	16	73,805,209	ZFHX3	Intronic	C	A	0.495	1.00E-06	0.573
2	rs6009715	22	49,351,462	-	Intergenic	G	A	1.802	2.30E-05	0.559
3	rs66598464	4	100,323,833	ADH1C; ADH17; ENSG00000299279 lncRNA	Intronic	G	A	2.126	2.75E-05	0.230
4	rs711516	7	109,374,419	ENSG00000308019 lncRNA	Intronic	C	T	2.193	2.95E-05	0.194
5	rs11814051	10	121,283,241	RGS10	Intronic	C	T	0.375	2.98E-05	0.885
Top variants from the post-imputation GWAS										
1	rs12598508	16	73,805,025	ZFHX3	Intronic	C	T	2.11	4.9E-07	0.424
2	rs1511924	16	73,805,209	ZFHX3	Intronic	A	C	2.08	5.14E-07	0.423
3	rs711489	7	109,323,256	ENSG00000308019	Intronic	T	G	2.5	7.33E-06	0.189
4	rs711479	7	109,313,305	ENSG00000308019	Intergenic	G	A	2.46	8.63E-06	0.190
5	rs10953946	7	121,403,829	ENSG00000227743	Intronic	T	G	0.44	1.04E-05	0.211

Abbreviations: EAF, effect allele frequency.

### 3.4 Secondary gene-level and knowledge-guided prioritization analyses

Secondary analyses were performed separately using 325,686 unique array-based autosomal SNPs and 6,668,741 post-imputation autosomal SNPs. Two complementary methods MAGMA and KGWAS were successfully completed, whereas no stable Bonferroni-significant gene or knowledge guided signal was observed.

Figure 2 shows the results of MAGMA analysis using the Kazakh in-sample LD reference panel for the array-based and post-imputation datasets. These results demonstrate that MAGMA gene ranking showed limited concordance between the two datasets. Genes nominally prioritized in the array-based MAGMA approach (Figure 2A) were not consistently retained after imputation (Figure 2B). Therefore, MAGMA results were interpreted as exploratory and were not used to define additional loci beyond the SNP-level GWAS findings. Additional MAGMA results using other LD reference panels are provided in Supplementary Figures S4, S5.

**FIGURE 2:**
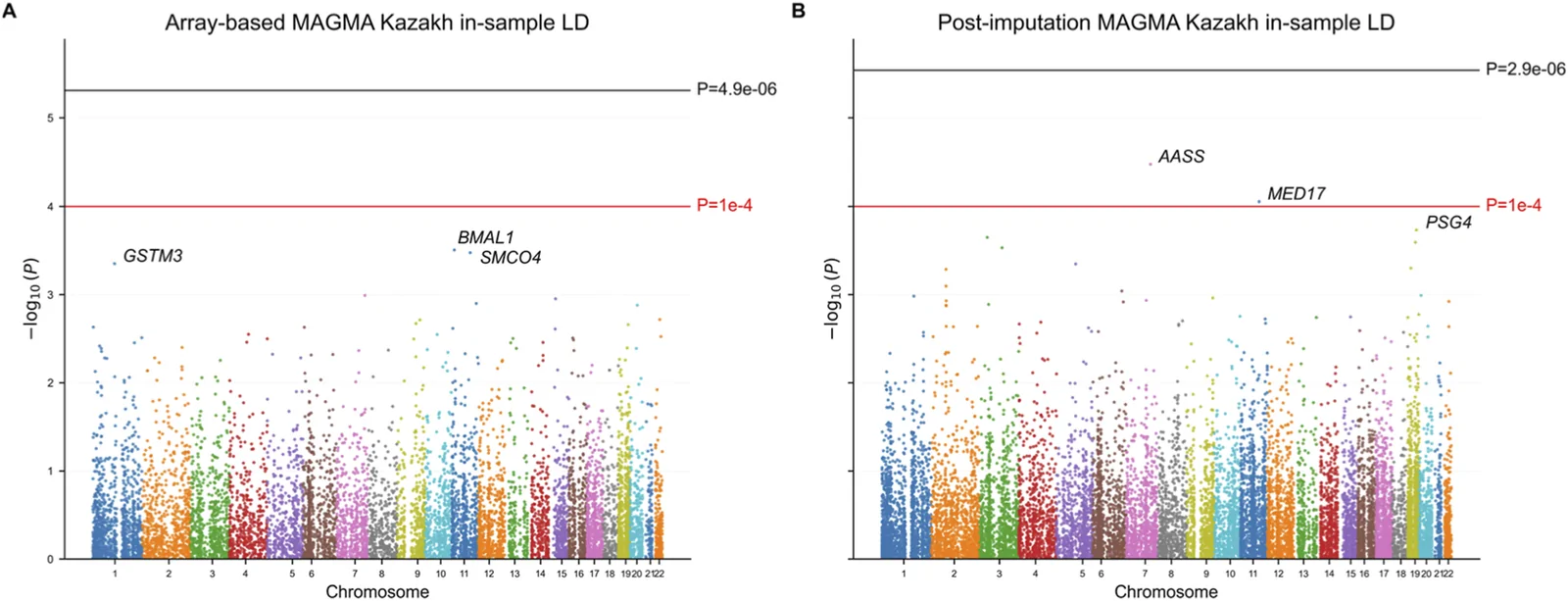
MAGMA results using the Kazakh in-sample LD reference. Gene-based Manhattan plots of **(A)** the MAGMA approach based on array-derived GWAS summary statistics in the Kazakh array-based in-sample LD reference; **(B)** the MAGMA approach based on post-imputation GWAS summary statistics in the Kazakh imputed in-sample LD reference. Each point represents a gene positioned at the midpoint of its annotated genomic interval and colored by chromosome. The y-axis shows −log_10_ of the MAGMA gene p-value. The upper horizontal black line indicates the Bonferroni-corrected genome-wide significance threshold. The horizontal red line marks the suggestive threshold.

KGWAS analysis was additionally performed as a knowledge-guided SNP-level prioritization analysis in repeated training runs. However, the analysis was limited by variant overlap with the KGWAS model space: only 82,033 array-based SNPs and 399,488 post-imputation SNPs were represented in the KGWAS output. The leading conventional GWAS variants in the ZFHX3 region, including rs1511924 and rs12598508, were not represented in the KGWAS output and therefore could not be directly evaluated using this approach. The top-ranked KGWAS-prioritized variants were not consistently reproduced across runs or between the array-based and post-imputation datasets (Supplementary Figures S6, S7). Accordingly, KGWAS results were also interpreted as exploratory and were not used to define additional loci.

In summary, this pilot GWAS of the IHD-HTN phenotype in the Kazakh cohort did not identify the Bonferroni-significant associations. However, the strongest SNP signal mapped to the ZFHX3 locus and was supported by the post-imputation analysis, supporting this locus as the main finding for the cohort studied.

## 4 Discussion

Cardiovascular genetics is increasingly interpreted within a population-specific framework. Although many genetic determinants of IHD, hypertension, and related cardiovascular phenotypes are shared across populations, differences in allele frequencies, LD structure, effect-size estimates, and gene–environment context may influence the detection and interpretation of risk loci, particularly for variants with modest effects. This is especially relevant because GWAS of coronary artery disease (CAD) remains dominated by European ancestry cohorts, while ancestry-specific CAD loci have been reported in available multi-ancestry studies (Silva et al., 2023; Ruan et al., 2022).

These considerations are particularly important for ethnic Kazakhs. Population genetic studies indicate that Kazakhs represent an admixed Central Asian population positioned between East Eurasian, Central Asian/Siberian, and West Eurasian genetic components, rather than a simple extension of either European or East Asian reference groups (Narasimhan et al., 2019; Seidualy et al., 2020; Zhao et al., 2020; Kairov et al., 2022; Lei et al., 2024). In agreement with the previous population genetic studies, our joint PCA analysis with 1000 Genomes Phase 3 reference populations showed a broad distribution of the studied Kazakh individuals across the Eurasian genetic landscape. Within-cohort PCA did not demonstrate discrete internal genetic clusters, whereas projection together with external reference populations showed a continuous distribution along the genetic continuum connecting European and East Asian reference populations. This pattern reflects the complex population structure of Central Asian populations and emphasizes the importance of considering ancestry-related variation in genomic studies of Kazakh cohorts. Such continuous ancestry variation may influence allele frequency distributions and LD patterns within relatively small cohorts, further supporting the need for ancestry-aware analytical strategies and population-relevant LD reference resources. Consistent with the complex population structure observed in the PCA, genome-wide LD decay analysis not only showed broadly similar distance-dependent patterns between the Kazakh cohort and the EUR, EAS, and SAS reference populations but also revealed modest quantitative differences in pairwise LD. In the common-variant analysis (MAF ≥0.05), EAS showed slightly higher mean LD at short genomic distances, whereas the Kazakh cohort generally showed median LD values at or near the upper range of the reference populations, with modestly elevated median (*r* ^2^) persisting at longer distances. A sensitivity analysis using the GWAS-related MAF threshold of ≥0.03 showed a similar qualitative pattern. These findings suggest that the overall genome-wide LD decay profile of the Kazakh cohort is broadly comparable to that of major Eurasian reference populations while not being fully equivalent in the distribution of pairwise LD. Importantly, similarity in genome-wide LD decay does not exclude population-specific differences in local LD structure at individual loci, which may remain relevant for LD-dependent downstream analyses. The slightly elevated longer-range LD observed in the pooled Kazakh cohort should also be interpreted cautiously as it may partly reflect the admixed and genetically heterogeneous composition of the study population. Additionally, recent HLA-based analyses of the population in Kazakhstan reported population-specific haplotypes and LD patterns, thereby underscoring the distinctive genetic architecture of this population (Turganbekova et al., 2025). Addressing these challenges requires population-relevant genomic resources. However, Kazakh genomic data remain underrepresented in major human genome resources, and large publicly available Kazakh reference panels suitable for routine GWAS workflows are still lacking or only beginning to emerge (Seidualy et al., 2020). Consequently, limitations in population-matched reference data may affect variant coverage, imputation accuracy, and interpretation of association signals in Kazakh cohorts. In this context, the present study is a pilot GWAS focused on the IHD-HTN phenotype in ethnic Kazakhs and demonstrates the practical importance of developing population-specific genomic resources and ancestry-aware analytical strategies for cardiovascular genetic research.

The choice of the combined IHD-HTN phenotype was clinically motivated. IHD and hypertension are age-related conditions that frequently coexist in routine cardiovascular practice, particularly in middle-aged and older adults. A NHANES study showed that comorbid CAD and hypertension are measurable and increasing phenotypes in the general adult population, with affected individuals having a median age of approximately 69–71 years (Chen et al., 2022). This pattern is consistent with the broader concept of cardiovascular multimorbidity where older patients with CVD often present with coexisting chronic conditions rather than isolated disease entities. In our study, the case group was deliberately restricted to individuals aged 50–70 years. This range was selected to focus on a clinically common group of patients who are regularly encountered in medical practice and often require long-term management for both IHD and hypertension. Patients older than 70 years were not included because recruitment of comparable apparently healthy controls becomes more difficult at advanced ages, and the probability of additional chronic, inflammatory, and metabolic conditions increases. Therefore, the selected age range represented a practical compromise between clinical relevance, phenotype definition, and control group comparability.

However, the IHD-HTN phenotype remains heterogeneous. Some patients with IHD-HTN may also have obesity, dyslipidemia, and lifestyle risk factors such as smoking. In our dataset, detailed information on body mass index, lipid profile, and smoking was unavailable or incomplete for part of the cohort. These limitations prevented more detailed covariate adjustment and stratified analyses and may have reduced the precision of genetic association estimates. Therefore, the results should be interpreted as pilot findings for a clinically relevant but heterogeneous cardiovascular phenotype.

Against this background of population and phenotype complexity, the present study was designed as a pilot case–control GWAS to explore genome-wide genetic signals associated with the combined IHD-HTN phenotype in an ethnic Kazakh cohort. The analysis did not identify any variant surpassing the Bonferroni-corrected significance threshold. This result should be interpreted in the context of the modest sample size, the polygenic architecture of cardiovascular traits, and the clinical heterogeneity of the combined phenotype. IHD with co-occurring arterial hypertension is likely influenced by multiple biological pathways, including vascular remodeling, blood pressure regulation, lipid and metabolic processes, inflammation, endothelial function, and environmental exposures. Therefore, the present GWAS should be considered a pilot discovery analysis aimed at identifying candidate signals for future validation rather than definitive locus discovery.

The strongest association signal was observed for rs1511924 at chromosome 16 within the ZFHX3 locus. The broader ZFHX3 locus has previously been implicated in cardiovascular phenotypes through variants other than rs1511924, including associations with atrial fibrillation and ischemic/cardioembolic stroke in populations of European and East Asian ancestry (Gudbjartsson et al., 2009; Li et al., 2011; Liu et al., 2014). Although rs1511924 did not reach the Bonferroni significance threshold, the prior cardiovascular relevance of the broader locus and the consistent regional signal after imputation support consideration of ZFHX3 as a hypothesis-generating candidate region requiring independent replication. Although imputation did not elevate this locus to the genome-wide significance, the post-imputation analysis provided additional regional support for the ZFHX3 signal. Regional LD analysis showed that the imputation variant rs12598508 was in nearly complete LD with the array-based lead variant rs1511924 (*r* ^2^ = 0.997), indicating that both variants likely represent the same underlying association signal. However, the absence of a broader genome-wide significant association pattern in the directly genotyped dataset and the lack of independent replication require cautious interpretation of this finding. Thus, the ZFHX3 region emerged as the strongest suggestive candidate locus identified in the present pilot dataset.

Because no individual variant reached the genome-wide significance threshold, we additionally evaluated whether gene-level and knowledge-guided prioritization approaches could identify aggregated or biologically supported signals that were not apparent at the single-variant level. However, secondary MAGMA and KGWAS analyses did not provide stable evidence for additional loci.

The interpretation of gene-based analyses in underrepresented populations is influenced by multiple factors, including GWAS power, SNP coverage, gene annotation strategies, and the availability of ancestry-matched LD reference panels. The population structure analysis performed in this study demonstrated substantial genetic diversity in the Kazakh cohort within the broader Eurasian context. This finding highlights the importance of population-relevant LD resources as local LD patterns may differ between populations even when overall genome-wide LD decay is broadly similar. Although we generated and applied an in-sample Kazakh LD reference panel for MAGMA, this resource was based on a modest number of individuals and cannot replace a comprehensive population-scale Kazakh LD reference panel. Therefore, the absence of stable MAGMA signals should be interpreted cautiously and may partly reflect limitations in available population-specific genomic resources rather than the absence of biologically relevant gene-level associations. Larger Central Asian cohorts together with ancestry-matched LD reference datasets will be required to improve the resolution of gene-based association analyses.

KGWAS was designed to enhance variant prioritization in small-cohort GWAS by integrating prior biological knowledge. However, in the present analysis, its application was limited by incomplete overlap between study variants and the KGWAS model space. Only 82,033 array-based and 399,488 post-imputation SNPs were represented in the KGWAS output, and the leading ZFHX3 variants identified in the conventional GWAS were not included. Consequently, KGWAS results were interpreted as exploratory prioritization analyses rather than confirmatory evidence for additional loci.

## 5 Limitations

The main limitations of this study include the modest sample size, clinical heterogeneity of the IHD-HTN phenotype, and the absence of an independent replication cohort. Because all cases had both IHD and hypertension, the present design does not permit phenotype-specific attribution of the observed genetic signals; accordingly, the findings should be interpreted in relation to the combined IHD-HTN phenotype. No independent cohort with a closely matched IHD-HTN case definition was available for replication. Although large public GWAS exists for IHD/CAD and hypertension separately, these phenotypes do not reproduce the combined case definition used here, and independent validation in a comparably defined cohort therefore remains necessary. Incomplete information on lifestyle, metabolic, and treatment-related covariates limited more detailed adjustment and stratified analyses. Secondary MAGMA and KGWAS analyses were additionally constrained by SNP coverage, variant representation, and the limited availability of Kazakh-specific LD reference resources.

## 6 Conclusion

In conclusion, this pilot GWAS did not identify genome-wide significant associations for the IHD-HTN phenotype in the Kazakh cohort. However, the ZFHX3 region emerged as the strongest suggestive candidate locus, supported by post-imputation and regional LD analyses. Joint population structure analysis revealed complex genetic structure within the studied Kazakh cohort and emphasized the importance of ancestry-aware approaches and population-specific genomic resources for cardiovascular genetic studies in Central Asia. To our knowledge, this study represents one of the first GWAS investigations of the IHD-HTN phenotype in ethnic Kazakhs and provides a foundation for future larger-scale genomic studies.

## Data Availability

Genome-wide association summary statistics generated in this study have been submitted to the NHGRI-EBI GWAS Catalog. Array-based GWAS summary statistics are available under accession GCST90841470 and post-imputation GWAS summary statistics under accession GCST90992880. Individual genotype and phenotype data are not publicly available because the informed consent and ethics approvals do not permit open sharing of individual genetic data. Additional analysis files and scripts are available from the corresponding author upon reasonable requests, subject to institutional and ethical restrictions.
