# Supplementary material for: A pilot genome-wide association study of ischemic heart disease with co-occurring arterial hypertension in a Kazakh cohort
Source: Front Genet. 2026 Oct 1;17:1927147. doi: 10.3389/fgene.2026.1927147 (PMC13668824; doi:10.3389/fgene.2026.1927147)
Supplement: Supplementary file 1 [file DataSheet1.docx]

**Supplementary Figure S1. Principal component analysis of the Kazakh study cohort and its population structure relative to 1000 Genomes reference populations.**

(A) Within-cohort principal component analysis of 470 Kazakh participants according to case/control status. Each point represents one individual colored by phenotype. Cases and controls showed substantial overlap in the PC1–PC2 space, with no clear phenotype-specific separation. (B) Joint principal component analysis of the Kazakh cohort together with European (EUR), East Asian (EAS) and South Asian (SAS) reference populations from the 1000 Genomes Project Phase 3. Kazakh individuals showed a broad distribution across the Eurasian PCA space, including positions overlapping or adjacent to the European and East Asian reference groups as well as intermediate positions.

**Supplementary Figure S2. Linkage disequilibrium decay in the Kazakh cohort and 1000 Genomes reference populations.**

Genome-wide linkage disequilibrium (LD) decay was compared between the Kazakh cohort (KAZ) and European (EUR), East Asian (EAS), and South Asian (SAS) reference populations from the 1000 Genomes Project Phase 3. (A) LD decay for common variants with MAF ≥0.05. (B) LD decay using the MAF ≥0.03 threshold corresponding to the array-based GWAS variant filtering. For each MAF threshold, variants passing the frequency criterion within each population were restricted to the common intersecting autosomal SNP set across KAZ, EUR, EAS, and SAS. Pairwise LD ((r^2)) was calculated for SNP pairs located within 1 Mb without prior LD pruning and summarized across physical-distance bins. Left panels show mean pairwise (r^2), whereas right panels show exact median pairwise (r^2). The x-axis represents physical distance between SNPs on a logarithmic scale. Overall, the four populations showed similar distance-dependent LD decay, with modest quantitative differences in LD magnitude between the Kazakh cohort and the reference populations.

**Supplementary Figure S3. Regional association plot of the ZFHX3 locus based on post-imputation GWAS results.**

Regional association plot of the chromosome 16 region surrounding rs1511924 (±500 kb) based on the post-imputation GWAS of IHD-HTN in the Kazakh cohort. Each point represents an imputed variant and colors indicate linkage disequilibrium (r²) with the lead variant rs1511924 calculated using the Kazakh in-sample imputed genotype reference panel. The lead array-based variant rs1511924 and the top post-imputation signal rs12598508 are highlighted. The lower panel shows gene annotations from GENCODE v50 (GRCh38), including the ZFHX3 and ZFHX3-AS2 loci. The dashed and dotted horizontal lines indicate the conventional genome-wide significance threshold (P = 5 × 10⁻⁸) and suggestive significance threshold (P = 1 × 10⁻⁵), respectively.

**Supplementary Figure S4. MAGMA gene-level analysis on array-derived GWAS summary statistics using East Asian and European linkage disequilibrium reference panels.**

Manhattan plots are shown for MAGMA analyses using (A) East Asian (EAS) linkage disequilibrium (LD) reference panel and (B) European (EUR) LD reference panel. Each point represents one gene plotted according to the genomic position. The red line indicates P = 1 × 10^-4^ and the black line indicates the Bonferroni-corrected threshold. The EAS and EUR panels were selected as ancestrally relevant proxy references because Kazakh population carry both East Eurasian and West Eurasian genetic components. These analyses were performed as comparative sensitivity analyses to evaluate the dependence of gene-level prioritization on LD reference choice given the limited availability of publicly available Kazakh LD reference panel.

**Supplementary Figure S5. MAGMA gene-level analysis on post-imputation GWAS summary statistics using East Asian and European linkage disequilibrium reference panels.**

Manhattan plots are shown for MAGMA analyses using (A) East Asian (EAS) linkage disequilibrium (LD) reference panel and (B) European (EUR) LD reference panel. Each point represents one gene plotted according to the genomic position. The red line indicates P = 1 × 10^-4^ and the black line indicates the Bonferroni-corrected threshold. The EAS and EUR panels were selected as ancestrally relevant proxy references because Kazakh population carry both East Eurasian and West Eurasian genetic components. These analyses were performed as comparative sensitivity analyses to evaluate the dependence of gene-level prioritization on LD reference choice given the limited availability of publicly available Kazakh LD reference panel.

**Supplementary Figure S6. KGWAS SNP-level prioritization across repeated training runs.**

Manhattan plots show KGWAS prioritized SNP-level results from three independent training runs based on the array-based GWAS summary statistics. A – the first independent KGWAS training run; B – the second independent KGWAS training run; C – the third independent KGWAS training run. Each point represents one SNP plotted according to the genomic position with the y-axis showing -log_10_(KGWAS P-value). The red horizontal line indicates the suggestive threshold of P = 1 × 10^-5^ and the black horizontal line indicates the Bonferroni-corrected threshold for the KGWAS variant dataset. No variant reached the Bonferroni-corrected threshold and top ranked variants were not consistently reproduced across repeated runs indicating the limited stability of KGWAS prioritization in this dataset.

**Supplementary Figure S7. Imputed** **KGWAS SNP-level prioritization across repeated training runs.**

Manhattan plots show KGWAS prioritized SNP-level results from the three independent training runs based on the post-imputation GWAS summary statistics. A – the first independent KGWAS training run; B – the second independent KGWAS training run; C – the third independent KGWAS training run. Each point represents one SNP plotted according to the genomic position with the y-axis showing -log_10_(KGWAS P-value). The red horizontal line indicates the suggestive threshold of P = 1 × 10^-5^ and the black horizontal line indicates the Bonferroni-corrected threshold for the KGWAS variant dataset. No variant reached the Bonferroni-corrected threshold and top ranked variants differed across repeated runs indicating the limited stability of KGWAS prioritization after the imputation.
